# The relevance of basic numerical skills for fraction processing: Evidence from cross-sectional data

**DOI:** 10.1371/journal.pone.0281241

**Published:** 2023-01-31

**Authors:** Silke M. Wortha, Elise Klein, Katharina Lambert, Tanja Dackermann, Korbinian Moeller

**Affiliations:** 1 LEAD Graduate School & Research Network, University of Tuebingen, Tuebingen, Germany; 2 Department of Neurology, University Medicine Greifswald, Greifswald, Germany; 3 Centre for Mathematical Cognition, School of Science, Loughborough University, Loughborough, United Kingdom; 4 University of Paris, LaPsyDÉ, CNRS, Paris, France; 5 Leibniz-Institut für Wissensmedien, Tuebingen, Germany; 6 University of Education, Heidelberg, Germany; 7 Individual Development and Adaptive Education Center, Frankfurt am Main, Germany; Carnegie Mellon University, UNITED STATES

## Abstract

Recent research indicated that fraction understanding is an important predictor of later mathematical achievement. In the current study we investigated associations between basic numerical skills and students’ fraction processing. We analyzed data of 939 German secondary school students (age range = 11.92 to 18.00 years) and evaluated the determinants of fraction processing considering basic numerical skills as predictors (i.e., number line estimation, basic arithmetic operations, non-symbolic magnitude comparison, etc.). Additionally, we controlled for general cognitive ability, grade level, and sex. We found that multiplication, subtraction, conceptual knowledge, number line estimation, and basic geometry were significantly associated with fraction processing beyond significant associations of general cognitive ability and sex. Moreover, relative weight analysis revealed that addition and approximate arithmetic should also be considered as relevant predictors for fraction processing. The current results provide food for thought that further research should focus on investigating whether recapitulating basic numerical content in secondary school mathematics education can be beneficial for acquiring more complex mathematical concepts such as fractions.

## Introduction

It has been argued that children’s numerical development is driven by the acquisition of basic numerical skills (e.g., [1, 2]). These basic numerical skills are seen as building blocks for later numerical and mathematical achievement. For successful numerical development different basic numerical skills were found to be important: For instance, symbolic and non-symbolic magnitude knowledge (e.g., [3–5]), as well as a spatial representation of magnitudes as described by the metaphor of the mental number line (e.g., [6, 7]), understanding of the place-value structure of the Arabic number system (e.g., [8, 9]), acquisition of arithmetic fact knowledge (i.e., multiplication tables; [3]), as well as skills on procedural and conceptual numerical knowledge (e.g., carry operations, or understanding of the relationship between addition and multiplication [10, 11]).

Therefore, it comes with no surprise that the mastery of such basic numerical skills predicts not only future numerical skills and mathematical achievement in school (e.g., [6, 8]), but also more general life prospects (i.e., employment rate; e.g., [12–14]). With regard to educational / mathematical achievement, several studies indicated that mastery of certain basic numerical skills were found to be associated with later mathematical achievement: For instance symbolic and non-symbolic magnitude knowledge (e.g., [15–18])and understanding the place-value structure of the Arabic number system [8]. Therefore, basic numerical skills are seen as highly relevant for children’s development of typical numerical and arithmetical capabilities.

Moreover, not only basic numerical skills are critical for children’s numerical development, but also the mastery of more advanced mathematical skills like handling fractions and the ability to successfully calculate with fractions are important steps in children’s mathematical development especially in secondary school [19, 20]. Accordingly, there is evidence that students’ fraction knowledge is a valid predictor of their actual but also future math achievement. For instance, [21] found that children’s early understanding of fractions in 5th or 6th grade predicted their later mathematical achievement and knowledge of algebra in high school even when controlling for IQ, reading achievement, working memory, family education and income, and whole number arithmetic knowledge (see also [22, 23]). As such, understanding fractions provides a critical foundation for later algebra learning (e.g., [24]).

In addition, several studies investigated the relevance of domain-general and domain-specific skills for fraction magnitude knowledge and fraction arithmetic (e.g., [23, 25–33]). For instance, [25] observed that knowledge of whole number magnitude and arithmetic in 1^st^ grade predicted knowledge of fraction magnitude and arithmetic in middle school (i.e., 7^th^ and 8^th^ grade) even after controlling for general cognitive abilities, parental education, parental income, race, and gender. Interestingly, however neither whole number magnitude nor whole number arithmetic predicted reading achievement in middle school. Moreover, in another longitudinal study [26] observed that number line estimation, non-symbolic proportional reasoning, division, working memory, and attentive behavior contributed to 6^th^ graders’ knowledge of fraction concepts, whereas number line estimation, multiplication fact fluency, division, and attention contributed to knowledge of fraction procedures.

Moreover, [33] found that basic numerical skills such as magnitude understanding and calculation fully mediated the association between general cognitive abilities (e.g., attention, working memory, etc.) in 3^rd^ grade and fraction knowledge in 6th grade. Finally, [28] evaluated developmental predictors of fraction concepts and fraction procedures in school children. The authors observed that attentive behavior, language, non-verbal reasoning, number line estimation, calculation fluency and reading fluency predicted conceptual understanding of fractions; while attentive behavior, number line estimation, calculation fluency and working memory predicted procedural understanding of fractions.

Taken together, previous studies identified several (basic) numerical skills and domain-general predictors as relevant predictors for later numerical and arithmetical performance including fractions, while understanding fractions predicts more advanced mathematical and algebraic achievement.

The current study aimed at investigating in more depth which basic numerical skills are associated with fraction processing as potential building blocks of fraction learning in German secondary school students using a cross-sectional design. We hypothesized that basic numerical skills reflecting the understanding of number magnitudes but also of basic arithmetic operations should be particularly relevant to fraction processing. As regards other basic numerical skills such as conceptual knowledge or basic geometry our approach was exploratory. Specifically, we evaluated associations between basic numerical skills and fraction processing in a two-step approach. Similar to previous studies, in the first step we aimed at identifying basic numerical skills significantly predicting fraction processing using multiple regression analysis. However, going beyond previous studies, we performed a relative weights analysis in the second step to assess the relative contributions of correlated predictors, as contributions from individual predictors may be masked due to shared variance. In doing so, we aimed to consider issues of multicollinearity between predictor variables in multiple regression analysis, which can be expected when predictor variables measure similar (e.g., natural number magnitude and fraction magnitude) or related concepts (e.g., approximate number system and mathematics achievement). This is often the case in studies that aim to determine predictors of math achievement in general (e.g., [34]), but also fraction processing in particular (e.g., [33]). This can lead to underestimation of the relevance of certain predictor variables, because (multiple) regression analysis might fail to indicate their significance as the variable shares too much variance with another significant predictor variable. As such, relative weight analysis determining the relative contribution of predictor variables to explained variance in the criterion independent of the predictors being significant or not might offer valuable new insights into the predictors of fraction processing over and beyond results of previous studies employing regression analysis. Taken together, the aim of the current study was to identify and differentiate associations between basic numerical skills and fraction processing and to quantify the relative importance of predictor variables.

## Methods

### Participants

For this study a subsample of *N* = 1248 students from German schools in the federal state Baden-Württemberg was analyzed. We included only children from 7^th^ to 11^th^ grade as introduction to fractions occurs at the end of 6^th^ grade in the state’s mathematics curriculum. Consistent with the state’s mathematics curriculum, by the end of 6^th^ grade students should be able to master different conceptual aspects of fractions including part-whole relations, measurement, density, and equivalency, but also more procedural aspects such as fraction arithmetic and converting fractions into decimals and vice versa. We excluded students older than 18 (likely repeaters; *N* = 272) and students with missing values on at least one of the considered variables (*N* = 37, i.e., the tests on basic numerical skills, grade level, sex, or general cognitive ability), resulting in a final sample of 939 students (7^th^ grade *N* = 200, 8^th^ grade *N* = 215, 9^th^ grade *N* = 210, 10^th^ grade *N* = 136, 11^th^ grade *N* = 178) for the analyses (age *M* = 15.14 years, *SD* = 1.49; 47% females). The current work presents parts of a larger project in which a standardized test of basic numerical as well as curricular mathematical abilities for secondary school was developed. Please note that secondary school in Germany starts as early as 5^th^ grade and can go up to 12^th^ or 13^th^ grade covering an age range from about 11 to 18 years on average. To test our hypotheses we used a statistical approach similar to [35, 36].

### Measures

#### Basic numerical skills

A battery to assess different basic numerical skills was administered including eight subtests to be completed in the following order: i) addition, ii) subtraction, iii) multiplication, iv) number line estimation, v) approximate arithmetic, vi) conceptual knowledge about arithmetic principles, vii) non-symbolic magnitude comparison, and viii) basic geometry. All subtests had a time limit to assess the level of automatization and because of test economy. Furthermore, all subtests only addressed numerical/mathematical skills of primary school curriculum and examples to ensure task understanding. Unless indicated differently, correctly solved items were considered as sum scores for analyses. In the following, the respective subtests are described separately and in more detail. Additionally, common examples for items of the different tests are given in Appendix A in S1 Appendix.

*Arithmetic operations*. These included i) *addition*, ii) *subtraction* and iii) *multiplication* with 36 arithmetic problems each, ordered in increasing difficulty. Addition and subtraction problems covered numbers ranging up to 10,000. Multiplication problems covered problems with single-digit, two-digit and three-digit operands (with a maximum problem size of 72, 4698, 3400, respectively). For each operation, students had to solve as many problems as possible within 2 minutes. In all three arithmetic operation tests, the sum of correctly solved items served as predictor variable, respectively.

*Number line estimation*. Students had to estimate the correct location of a given number on a number line. Only the endpoints of the number line were defined (e.g., estimate the location of 64 on a number line ranging from 0 to 100). In total, the task included 24 items with changing endpoints (i.e., 6 items on a number line from 0 to 10 and 0 to 100, respectively, and 4 items on a number line from 0 to 1.000, 0 to 10.000 and 0 to 100.000, respectively). To prevent counting-based strategies, there was a time limit of 1.5 minutes. The mean percentage absolute estimation error (PAE; cf. [37]) served as predictor variable. Items with no response were not considered for analyses.

*Approximate arithmetic*. Students were presented a problem with two different incorrect solution probes. They were instructed to solve the task by estimating and choosing the solution being closer to the correct result (e.g., “Which result is closer to 347–120? solution probes: 215 or 260”). 16 addition and 16 subtraction problems were presented, whose difficulty level increased with item number. Students had 2 minutes to solve as many problems as possible. The sum of correctly solved items served as predictor variable.

*Conceptual knowledge about arithmetic principles*. Students were shown two problems of which the first problem was presented with a solution. They were instructed to decide whether the solution of the first problem helped to solve the second problem without having to calculate (e.g., “Does 4 + 8 = 12 help you to solve 12–4 = __?”). 40 pairs of arithmetic problems including addition, subtraction, multiplication, and division were presented (i.e., 20 problems in which the first equation was helpful to know for solving the second one and 20 in which this was not the case). Students were instructed to make a correct decision for as many problems as possible within 2 minutes. This test required students to correctly identify relationships between numerical/arithmetic operations. This should be possible without actually solving the arithmetic problems involving the corresponding calculations. The sum of correctly solved items was used as predictor variable.

*Non-symbolic magnitude comparison*. 24 pairs of dot clouds (ranging from 30 to 69) were shown, and students had to decide which of the two dot clouds was numerically larger. Dot clouds were matched for overall surface to prevent children from using strategies based on perceptual features: for half of the items the surface with the lower quantity of dots was larger and for the other half the surface with the higher quantity of dots was larger. To avoid counting-based strategies, the time limit for this task was 1 minute. The number of correctly solved items served as predictor variable.

*Basic geometry*. Students had to solve 12 mirror image problems. For this, a flipped geometrical form had to be drawn by mirroring a presented form on a given axis. For each correct line in a drawing, students were given one point. Scores for each item varied between 6 and 12 points. The percentage of correctly solved items served as predictor variable. We chose to include a basic geometry test because our aim was to cover numerical skills of the German primary school curriculum as comprehensively as possible. Similar basic geometry tasks have been used in curriculum-based math achievement tests for primary schools in Germany (e.g., DEMAT 3+, [38]; DEMAT 4, [39]).

#### General cognitive ability

Students’ general cognitive ability was assessed using two subtests of the German version of the Culture Fair Intelligence Scale 20-revision (i.e., continuation of sequences and completion of matrices; CFT 20-R; [40]). In the *continuation of sequences* subtest, students are given a sequence of changing shapes and they need to find a logical continuation to this sequence. In the *completion of matrices* subtest, students are given a matrix of changing shapes and they need to find a logically matching shape for the blank cell of the given matrix. Subtests were administered as described in the manual. The sum of correctly solved items served as predictor variable.

#### Fraction processing

Fraction processing was evaluated by assessing fraction arithmetic (eight items, e.g., calculate and simplify as far as possible: $\frac{1}{3}+\frac{5}{8}=?$) and fraction magnitude processing (four items, e.g., writing the proportion reflected by two differently shaded areas as a fraction (2 items) or converting one fraction into an equivalent fraction (2 items)). Fraction arithmetic items included addition (3 items), subtraction (2 items) and division (3 items) with unequal denominators. The test had a time limit of 3 minutes in total. Correctly answered items were considered in a sum score, which served as criterion variable in all analysis.

#### Procedure

All tests were administered during regular school hours in the students’ classrooms and testing took a maximum of 90 minutes. For students below the age of 18, parents received information about the study and provided written informed consent prior to testing, whereas students above the age of 18 provided written informed consent themselves. The study was approved by the local ethical committee (A2.5.4–42_sn) and the regional school authorities for adherence to ethical standards and data protection regulations. Students received instructions by trained student assistants. To ensure that children understood each task, written instructions were read to students to introduce them to each subtest. Additionally, examples were provided to make sure students understood the tasks in the respective subtests. All tests were time-limited for reasons of test economy and to assess automatization.

### Statistical analyses

#### Multiple regression

Multiple regression analysis was used to determine the associations between the measured basic numerical predictors and fraction processing. We used False Discovery Rate (FDR) controlling the *p*-value for multiple testing [41].

#### Relative weight analysis

Determining the relative contribution of each predictor to the explained variance is usually difficult. For instance, [42] pointed out that it is problematic to use multiple regression for assessing the relative importance of correlated predictors as this method is not able to correctly partition variance to the different correlated predictors. As predictors are usually correlated with one another, relative weight analysis is more appropriate to evaluate the relative importance of predictors. Therefore, we used relative weight analysis as suggested by [43] to quantify the relative importance of correlated predictor variables in the multiple regression analysis.

Relative weight analysis determines which variables contribute the most regarding explained variance in terms of *R*^*2*^. For this, the predictor variables are transformed into a set of orthogonal variables in a way that they are maximally related to the original predictors. The resulting relative weights represent the predictors’ additive decomposition of the total model *R*^2^. There are two measures of relative weight: *raw relative weight* and *rescaled relative weight*. Raw relative weights add up to the *R*^2^ of the model while rescaled relative weights add up to 100% representing the relative importance of a particular variable in the final regression model.

Relative weights can be understood as the share of declared variance to which each predictor variable can be appropriately assigned [44]. We identified significance of relative weights using the procedure described by [45]. Importantly, relative weight analysis not only helps evaluating how much variance each predictor explains by itself and in conjunction with other predictors. Instead, it is also able to uncover ulterior predictors that regression analyses may miss to detect due to shared variance of correlated predictors (see [46] for a more detailed introduction and discussion).

#### Variables

Overall, 11 predictor variables were incorporated into the multiple regression and relative weight analysis. These included the eight basic numerical skills assessed (i.e., addition, subtraction, multiplication, number line estimation, approximate arithmetic, conceptual knowledge about arithmetic principles, non-symbolic magnitude comparison, and basic geometry) as well as general cognitive ability, grade level and sex. All continuous variables and grade level were centered, and sex was effect coded (i.e., female = -1, male = 1) prior to analyses.

#### Statistical software

Statistical analysis was performed with R [47]. For the multiple regression analysis, we used the ‘lm’ function for fitting linear models of the standard R package “state” [47]. Additionally, we used the ‘p.adjust’ function with the ‘fdr’ method to adjust *p*-values. For the relative weights analysis, we applied the syntax provided by [44].

## Results

### Descriptive statistics

Mean fraction processing score was *M* = 3.31 (SD = 2.17, obtained range = 0–11) of 12 possible points. Descriptive statistics for all predictor variables are shown in Table 1. For detailed results of descriptive statistics separated by grades (i.e., 7^th^ grade to 11^th^ grade) see Appendix B Figures A1-A5 in S1 Appendix. Please note that results did not change substantially when using age instead of grade level as predictor in the analysis.

**Table 1:** Descriptive statistics and obtained range of all basic numerical measures (N = 939).

	*M*	*SD*	*Range*	*Max score*
Addition	20.06	4.13	2–31	36
Subtraction	16.38	4.71	0–32	36
Multiplication	20.92	4.25	4–29	36
Number line estimation (PAE)	6.52	4.07	1.58–47.47	-
Approximate arithmetic	19.62	5.12	0–32	32
Conceptual knowledge	18.12	6.18	2–36	40
Basic geometry (%)	58.23	21.17	0.00–100.00	100
Non-symbolic magnitude comparison	18.65	3.19	1–24	24
General cognitive ability	18.77	4.28	3–29	30

The correlation matrix depicted in Table 2 indicates that all basic numerical skills were significantly correlated with fraction processing, as well as amongst each other. The highest correlation between two basic numerical skills was *r* = .70 and was observed between subtraction and addition performance, followed by high correlations between multiplication and addition performance as well as multiplication and subtraction performance (*r* = .57, respectively). About 70% of correlations were below *r* = .30. Sex was negatively correlated with fraction processing indicating that females performed better in the fraction processing test than males (see Table 2).

**Table 2:** Correlations between fraction processing, basic numerical skills, general cognitive ability, as well as sex and grade level.

Variable	1	2	3	4	5	6	7	8	9	10	11
1. Fraction processing	1										
2. Addition	**.39** ^**^	1									
3. Subtraction	**.42** ^**^	**.70** ^**^	1								
4. Multiplication	**.42** ^**^	**.57** ^**^	**.57** ^**^	1							
5. Number line estimation	**-.25** ^**^	**-.14** ^**^	**-.25** ^**^	**-.16** ^**^	1						
6. Approximate arithmetic	**.24** ^**^	**.44** ^**^	**.48** ^**^	**.34** ^**^	**-.13** ^**^	1					
7. Conceptual knowledge	**.34** ^**^	**.38** ^**^	**.36** ^**^	**.33** ^**^	**-.12** ^**^	**.43** ^**^	1				
8. Basic geometry	**.27** ^**^	**.20** ^**^	**.22** ^**^	**.18** ^**^	**-.23** ^**^	**.07** ^*^	**.20** ^**^	1			
9. Non-sym. mag. comp.	**.08** ^*^	**.13** ^**^	**.12** ^**^	**.14** ^**^	**-.06** ^*^	**.12** ^**^	**.09** ^*^	**.08** ^*^	1		
10. G. cognitive ability	**.41** ^**^	**.34** ^**^	**.37** ^**^	**.31** ^**^	**-.32** ^**^	**.20** ^**^	**.28** ^**^	**.39** ^**^	**.20** ^**^	1	
11. SexEff^a^	**-.13** ^**^	.06	**.16** ^**^	.01	**-.08** ^*^	**.17** ^**^	**-.09** ^*^	**-.07** ^*^	-.01	-.06	1
12. Grade level	.02	**.12** ^**^	**.11** ^**^	-.04	**-.14** ^**^	**.13** ^**^	.05	.03	.01	**.11** ^**^	**.19** ^**^

Note

^**^p < .01

^*^p < .05. N = 939.

^a^Code female = -1, male = 1

### Multiple regression analysis

The final regression model explained 34% of the variance [*R^2^* = .34, *adj*. *R^2^* = .33, *F*(11, 927) = 42.38, *p* < .001]. Seven variables significantly predicted performance in the fraction processing test: general cognitive ability, number line estimation, subtraction, conceptual knowledge, multiplication, basic geometry and sex (see Table 3). Inspection of beta weights indicated that better general cognitive ability, subtraction, conceptual knowledge, multiplication, and basic geometry performance was associated with better performance on the fraction processing test. Additionally, smaller estimation errors in the number line estimation task predicted better performance on the fraction processing test.

**Table 3:** Multiple regression and relative weight results.

	*B*	β	[*L-CI*,*U-CI*]	*RW*	*t*	*p*	*RS-RW* (%)
Criteria = Fraction processing [multiple *R^2^ =* .34, *adj*.* R^2^* = .33, *F*(11,927) = 42.38, *p* < .001]							
General cognitive ability	0.09	.18	[.25, .53]	.06	5.53	.000	18.61*
Multiplication	0.09	.18	[.24, .54]	.06	5.09	.000	18.40*
Subtraction	0.07	.14	[.13, .49]	.05	3.43	.001	15.01*
Conceptual knowledge	0.04	.12	[.13, .40]	.04	3.83	.000	11.59*
Addition	0.03	.06	[-.04, .30]	.04	1.50	.178	11.51*
Number line estimation (PAE)	-0.06	-.11	[-.36, -.11]	.03	-3.69	.000	7.91*
Basic geometry	0.01	.07	[.02, .27]	.02	2.25	.037	6.92*
Sex^a^	-0.30	-.14	[-.42, -.18]	.02	-4.79	.000	5.56*
Approximate arithmetic	0.00	.01	[-.12, .16]	.01	0.23	.816	3.96*
Non-sym. mag. comp.	-0.02	-.03	[-.19, .04]	.00	-1.22	.268	0.29
Grade level	-0.02	-.01	[-.15, .09]	.00	-0.49	.683	0.23

We did not additionally analyze our data separately for grade levels, because grade level was not a significant predictor in our multiple regression and relative weight analysis. This indicates that the associations between basic numerical skills and fraction processing did not differ significantly across grade levels. Note: *B*: unstandardized regression weight; *β*: standardized regression weight; *L-CI*: lower boundary (2.5%); *U-CI*: upper boundary (97.5%); *RW*: raw relative weight (within rounding error raw weights will sum to R^2^); *t*: t-value measures the size of the effect relative to the variation in sample data; *p*: *p*-value FDR-corrected for multiple comparisons; *RS-RW*: relative weight rescaled as a percentage of predicted variance in the criterion variable attributed to each predictor (within rounding error rescaled weights sum to 100%).

^a^ code female = -1, male = 1.

* significantly different from a random variable.

### Relative weight analysis

We performed relative weight analysis to reveal the relative contribution of each predictor variable to the total variance of *R*^2^ while accounting for multicollinearity (e.g., [48, 49]).

Rescaled relative weights indicated that the proportional contribution of the predictor *general cognitive ability* explained 18.61% of the total variance in the fraction processing test. Considering basic numerical skills, multiple regression as well as relative weight analysis point to *multiplication* as the best predictor for fraction processing (18.40%). This was followed by *subtraction* (15.01%), *conceptual knowledge* (11.59%), *number line estimation* (7.91%), basic geometry (6.92%), and *sex* (4.91%).

Although, addition and approximate arithmetic were not found to be significant predictors in the multiple regression analysis, relative weight analysis revealed rescaled relative weights that were significantly different from a random variable (addition: *RS-RW* = 11.51%; approximate arithmetic: *RS-RW* = 3.96%). Therefore, these two basic numerical skills might also be relevant for the prediction of fraction processing.

## Discussion

In the present study, we investigated the association between secondary school students’ basic numerical skills and fraction processing. We aimed at evaluating our hypothesis that basic numerical skills reflecting the understanding of number magnitude and basic arithmetic operations should be particularly relevant to fraction processing (cf. [33]) in German secondary school students. Additionally, we wanted to explore the relative associations of other basic numerical skills such as conceptual knowledge or basic geometry. Therefore, we chose a two-step approach: in a first step, we identified significant predictors of fraction processing using multiple regression. In a second step, we then evaluated relative importance of individual basic numerical skills using relative weight analysis (cf. [43]) to determine the relative contribution of each identified predictor to the explained variance and to uncover ulterior predictors that multiple regression analyses missed to detect.

In the first step, the regression analysis revealed that *general cognitive ability* and *multiplication* were the most important predictors of fraction processing. Moreover, *subtraction*, *conceptual knowledge*, *number line estimation*, *basic geometry* and *sex* significantly predicted fraction processing in the sense that better performance on these basic numerical skills and female sex was associated with better performance in the fraction processing test. In addition to these significant predictors, relative weight analysis–as the second step–indicated considerable associations of *addition* and *approximate arithmetic*.

These findings are largely in line with previous findings of longitudinal studies: whole number magnitude knowledge (i.e., number line estimation) and whole number arithmetic knowledge (i.e., multiplication, subtraction and conceptual knowledge of arithmetic principles) were observed to be important predictors of fraction processing (e.g., [25, 26, 28, 33]). In the following, contributions of significant predictors as revealed by the multiple regression analysis, but also of potentially relevant predictors as indicated by the relative weight analysis will be discussed in more detail.

The most important (and consistently observed) predictor of fraction processing was *general cognitive ability*: better general cognitive ability predicted better fraction processing. General cognitive ability may be defined as a general mental capability in problem solving, abstract thinking, reasoning, planning, and comprehension of novel problems, but also learning from experience (e.g., [50–52]). To assess general cognitive ability, we used two subtests of the CFT-20-R [40] reflecting fluid intelligence. Fluid intelligence is assumed independent of experience and previously acquired knowledge. According to Cattell [50, 52] it should also not be influenced by educational level and other environmental factors. Both, the subtest *continuation of sequences* as well as the subtest *completion of matrices* capture the ability to recognize rules and relationships. Transferred to fractions it is also important to recognize and then apply correct strategies and rules (e.g., [53, 54]). Unfortunately, most students tend to apply arithmetic strategies incorrectly when it comes to fractions (e.g., [55, 56])–one of the reasons is wrongly generalizing knowledge on whole number arithmetic to fraction arithmetic (e.g., [53]). Additionally, in the curriculum of Baden-Württemberg fractions are introduced by the end of 6^th^ grade, but hardly used afterwards in math classes and fraction problems are often not a common part of the daily math routine in schools resulting in a crucial lack of practice for solving fraction problems. As such, the ability to systematically solve problems on a more abstract level might be useful for operating on fractions. This is in line with previous studies indicating that general cognitive abilities like intelligence and cognitive flexibility (as also assessed by our measure), but also others like working memory and attention are crucial for fraction processing [57, 58]. Additionally, these and the present results substantiate earlier claims that general cognitive abilities play a central role in math processing [59]. This is supported by further evidence suggesting that children with deficits in general cognitive abilities seem to rely on misconceptions like the whole number bias more than typically developing children [58] and may thus be more likely to have difficulties with fraction processing. Therefore, general cognitive ability may be an important predictor of fraction processing test performance.

In our study, the most important basic numeric predictor of fraction processing, as identified consistently in regression and relative weight analysis, was *multiplication*, with better multiplication performance predicting better fraction processing test performance. This seems reasonable as a large part of the fraction processing test consisted of fraction arithmetic problems, for which multiplication is the key operation. For fraction addition and fraction subtraction, multiplication procedures are required to find the common denominator and/or extend the numerators. Additionally, multiplication is also a key operation for solving other problems like converting a fraction into an equivalent fraction. Moreover, for multiplication or division of fractions it is necessary to multiply numerators and denominators with each other. Therefore, fluency with multiplication facts should help students to solve fraction problems independently of task type (i.e., magnitude comparison or a fraction arithmetic; cf. [31, 33, 60]). Apart from that, multiplication is assumed to be solved by arithmetic fact retrieval (e.g., [61, 62]) which is fast, efficient and less effortful than any other procedural strategy based on magnitude manipulations.

Moreover, *subtraction* was identified as a significant and relevant predictor of fraction processing in both regression and relative weight analysis. Better subtraction performance was associated with better fraction processing. This may indicate that, in addition to arithmetic fact retrieval, processes of magnitude manipulation also play an important role in dealing with fractions, because subtraction is considered to be the arithmetic operation relying most on magnitude manipulations [63–65]. As mentioned above, a considerable part of the fraction processing test involved fraction calculations. The two most common operations used while calculating with fractions are multiplication (as described above) and addition. However, *addition* was not a significant predictor of fraction processing in the regression analysis. One reason may be that subtraction and addition performance were highly correlated and variability was slightly more pronounced for subtraction as compared to addition performance (see Tables 1 and 2). Moreover, as already mentioned above subtraction is assumed to draw on magnitude manipulations more strongly than addition. Therefore, it is unlikely that addition may explain unique variance beyond that already captured by subtraction. This argument is corroborated by the relative weight analysis, in which addition explained a relevant proportion of the variance in fraction processing performance.

Additionally, students’ *conceptual knowledge* on arithmetic principles was a significant predictor of their fraction processing. Better performance on the conceptual knowledge test was associated with better fraction processing. This test required students to correctly identify relationships between numerical/arithmetic operations without actually solving the arithmetic problems involving corresponding calculations. Therefore, an arithmetic principles knowledge of the reciprocal relationships between different arithmetic operations was necessary to correctly solve the task. Such understanding is important as recent research indicated that students often tend to rely on their procedural mathematical knowledge without really understanding which arithmetic procedure is the correct one and why it is the correct one (e.g., [66]). For the case of fractions, this may easily lead to an erroneous application of arithmetic procedures. Interestingly, resulting strategy errors (e.g., wrongly applying procedures for whole numbers) are more common than execution errors (e.g., [23, 54]). Therefore, better conceptual knowledge may enable students to apply arithmetic procedures correctly because they have good understanding of their relationships across arithmetic procedures.

Furthermore, better *number line estimation* performance predicted better performance in the fraction processing test. Number line estimation is considered to assess students’ representation of number magnitude (e.g., [67]). For successful numerical development, it is essential to understand the concept that all numbers represent numerical magnitudes that are aligned in ascending order on a mental number line. According to [4] (see also [5, 68]), the mental number line is a dynamic structure that is capable of representing all kinds numerical magnitudes (i.e., whole numbers, negative numbers and rational numbers). Additionally, magnitude understanding reflects a universal characteristic that applies to all kinds of real numbers (cf. [68]). In this vein, several studies indicated that individual differences in whole number magnitude understanding as assessed by the number line estimation task predicted later differences in fraction magnitude knowledge (e.g., [25, 28, 33]). Therefore, representing the magnitudes of whole numbers adequately on a mental number line seems to be a building block for successful understanding of fraction magnitudes later on. This is in line with previous findings showing that whole number magnitude knowledge is indeed a building block of later fraction processing (e.g., [69]). Moreover, [68] also argue that fundamental understanding of number magnitude is essential to understand arithmetic procedures. This is in line with our results as large parts of our fraction processing test involved fraction arithmetic problems and better whole number magnitude understanding predicted better performance in fraction arithmetic problems.

Better performance in the *basic geometry* task was associated with better performance in the fraction processing task. Previous research showed that spatial skills, which would be needed for successful performance in basic geometry, also play a crucial role in magnitude representations which is in turn crucial for fraction processing. For instance, [70] argued that early spatial skills foster the development of magnitude knowledge in supporting the construction of a mental number line. Moreover, [71] were able to show that visual-spatial skills as required in the basic geometry task were associated to fraction problem solving.

Finally, *sex* was a significant predictor in our fraction processing test with females performing better than males. One reason might be that fraction processing in general and fraction arithmetic in particular requires successful application of procedures or strategies learnt at school with females known to be more successful in doing so than males [72–74].

However, when a predictor variable in a multiple regression analysis is not significant, this does not necessarily mean that it has no influence on the dependent variable. Therefore, we conducted relative weight analyses. The latter revealed that *addition* and *approximate arithmetic* additionally contributed substantially to *R*^*2*^ and should also be considered as relevant predictors. However, as these three predictors were correlated with other predictors and therefore shared parts of their variance with other predictors it is likely that they were not able to become significant predictors in the multiple regression analysis because they may not have provided significant incremental information. As mentioned above, *addition* performance was highly correlated with subtraction performance, which was included in the final regression model. Finally, *approximate arithmetic* was correlated significantly with subtraction and addition. This task requires confident and fast manipulation of magnitudes by relying on either addition or subtraction. Therefore, it is unlikely that performance on approximate arithmetic would explain unique variance over and above of what had already been explained by subtraction and/or addition.

### Limitations and perspectives

In summary, we observed that students’ fraction processing was predicted significantly by their basic numerical skills. In particular, performance in multiplication, subtraction, conceptual knowledge and number line estimation were identified as significant predictors of children’s fraction processing test performance beyond associations of sex and general cognitive ability. However, in contrast to previous studies (e.g., [25, 26, 28]), our study is based on cross-sectional data and thus does not provide any information about developmental changes in fraction processing. Nevertheless, grade level was not a significant predictor, neither in the multiple regression analysis nor in the relative weight analysis. This indicates that performance in our fraction processing test was not predicted significantly by the grade level of participants. This is well in line with previous findings that fractions are difficult to understand and handle for students, adults and even math teachers [75–78]. However, when interpreting these results, it is important to keep in mind that all tests were time restricted, which could have affected performance in general, as students might have made more errors and might have been unable to solve all presented problems. However, our results fit nicely with that of prior longitudinal studies showing that whole number arithmetic (i.e., multiplication and subtraction) and number line estimation are important predictors for fraction processing (e.g., [25]). Furthermore, going beyond previous studies, relative weight analysis indicated that relevant associations between basic numerical skills on fraction processing are not limited to predictors identified as significant in a multiple regression analysis. Instead, considering the relative contributions of variables when transformed in orthogonal predictors revealed that also non-significant regression predictors explained relevant shares of variance in the criterion variable. This suggests that previous studies may have underestimated associations between basic numerical skills and fraction processing when not taking into account intercorrelations of regression predictors in their analyses.

As such, one might speculate that strengthening whole number magnitude understanding and whole number arithmetic should provide children with a broader fundament for fraction understanding. As an educational implication, this may indicate that recapitulation of basic numerical content (typically acquired in primary school) in secondary school mathematics instruction might be beneficial for acquiring more complex mathematical concepts such as fractions. However, while this seems to be suggested by the significant associations of basic numerical skills observed in the current study, this was not tested in this study and thus requires future research evaluating potential influences of basic numerical instruction in secondary school on the acquisition of more complex mathematical concepts including fractions.

## Supporting information

S1 Appendix (DOCX)
