# Supplementary material for: The relevance of basic numerical skills for fraction processing: Evidence from cross-sectional data
Source: PLoS One. 2023 Jan 31;18(1):e0281241. doi: 10.1371/journal.pone.0281241 (PMC9888716; doi:10.1371/journal.pone.0281241)
Supplement: S1 Appendix — (DOCX) [file pone.0281241.s001.docx]

**Appendix**

**A.** Item examples for the different subtests and the given instructions of the basic numerical skills test.


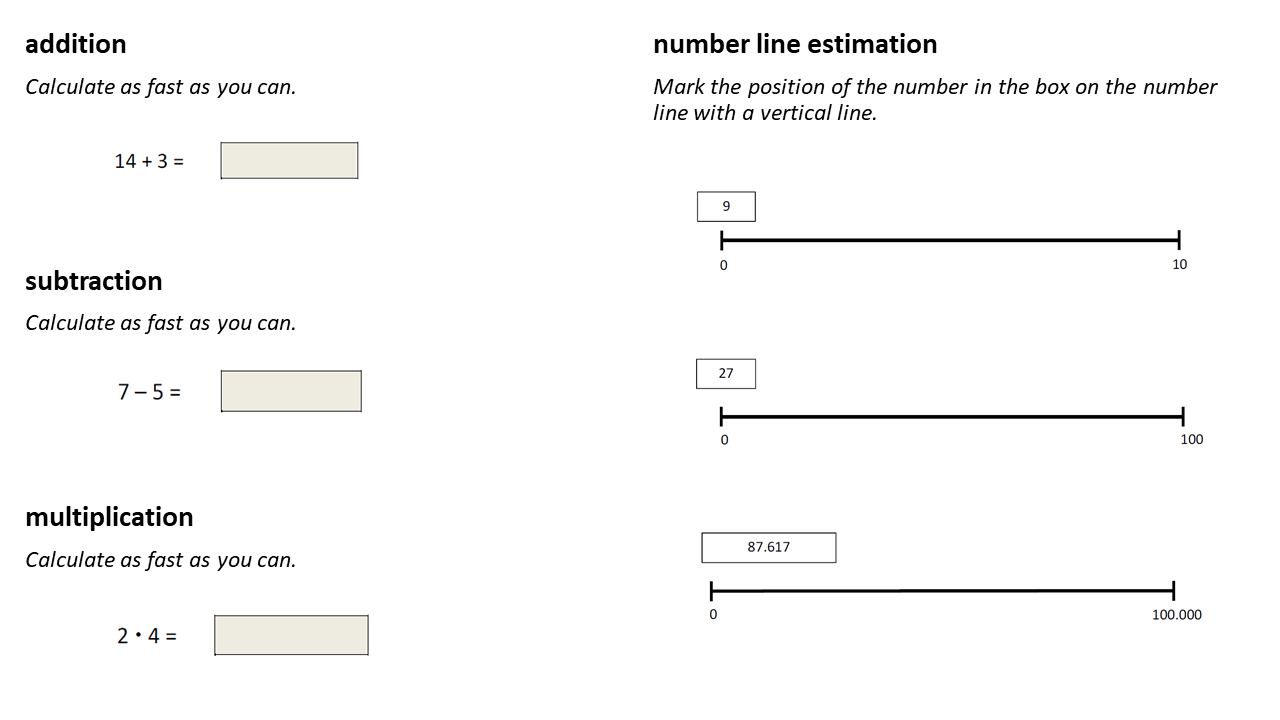


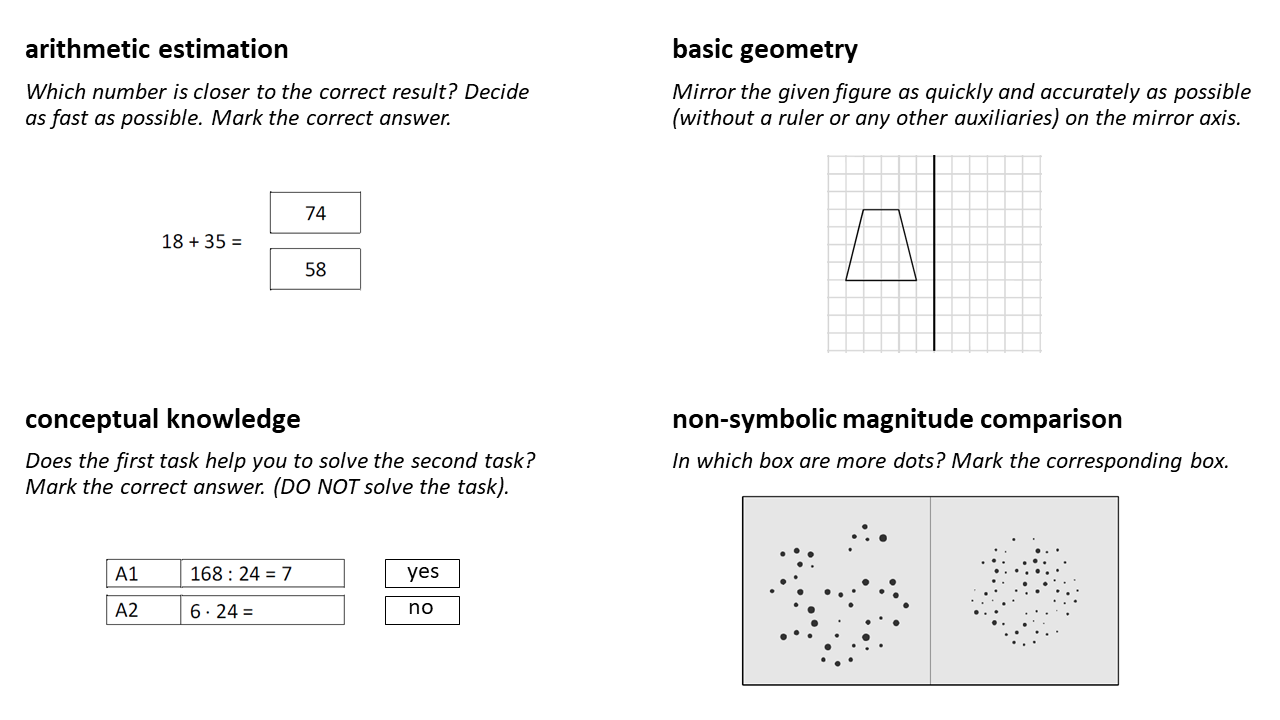


1. Descriptive Statistics of all variables of interest distinguished by grade levels (i.e., 5^th^ grade to 11^th^ grade).


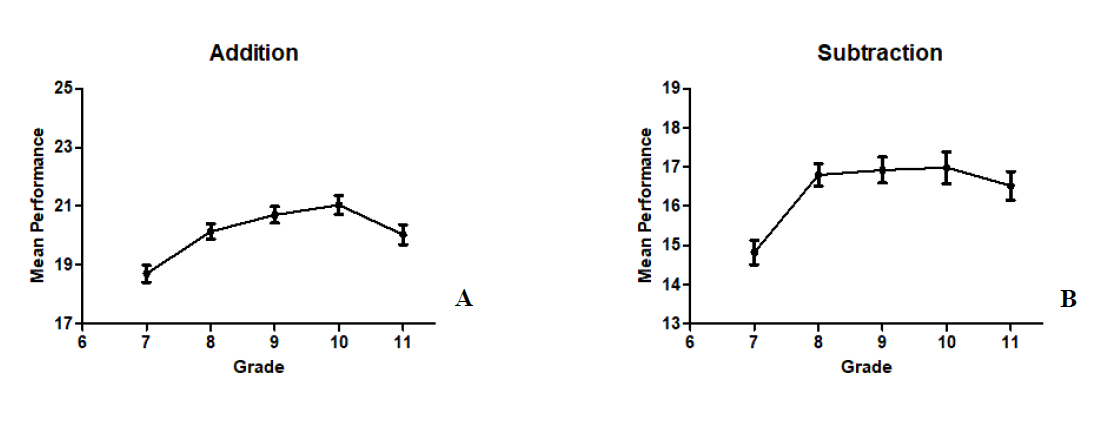


**Figure A1:** Students’ mean performance for addition **(A)** and subtraction **(B)** scales distinguished by grade levels (7^th^ grade *N* = 200, 8^th^ grade *N* = 215, 9^th^ grade *N* = 210, 10^th^ grade *N* = 136, 11^th^ grade *N* = 178).

**
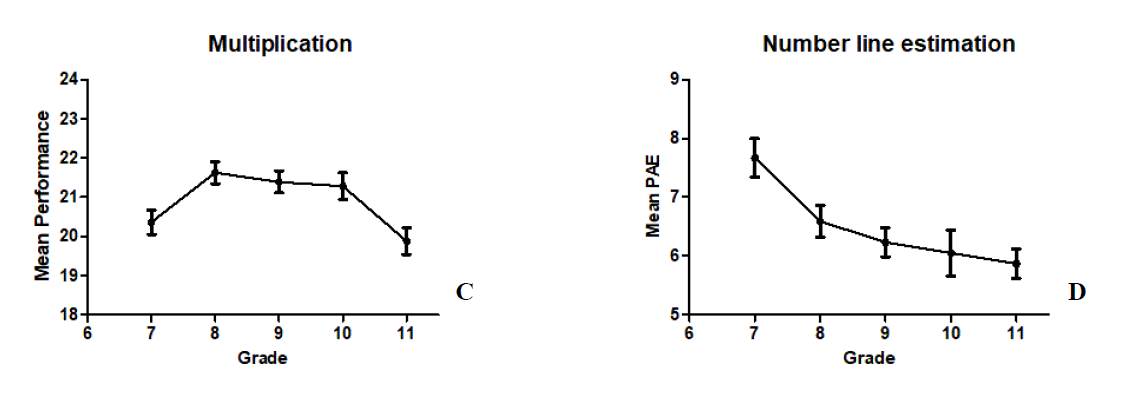
Figure A2:** Students’ mean performance for multiplication scale **(C)** and students’ mean percentage absolute estimation error for number line estimation scale **(D)** distinguished by grade levels (7^th^ grade *N* = 200, 8^th^ grade *N* = 215, 9^th^ grade *N* = 210, 10^th^ grade *N* = 136, 11^th^ grade *N* = 178).


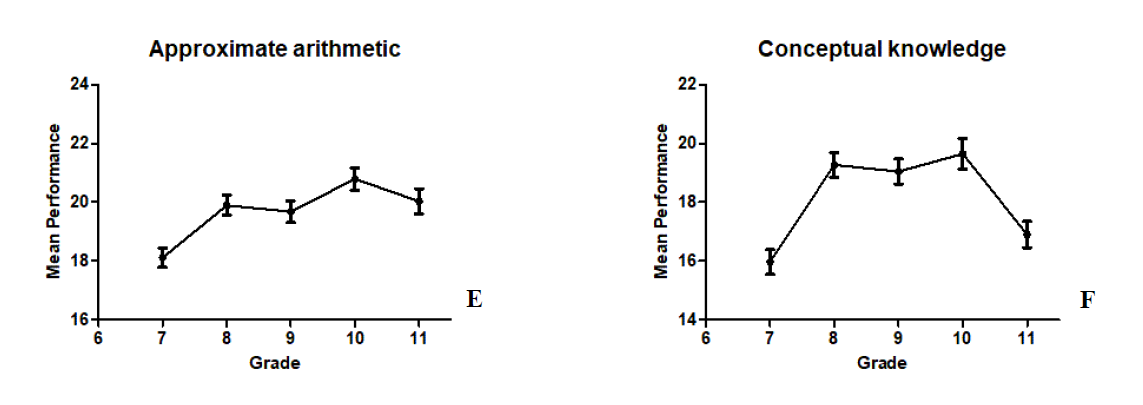


**Figure A3:** Students’ mean performance for approximate arithmetic **(E)** and conceptual knowledge **(F)** scales distinguished by grade levels (7^th^ grade *N* = 200, 8^th^ grade *N* = 215, 9^th^ grade *N* = 210, 10^th^ grade *N* = 136, 11^th^ grade *N* = 178).


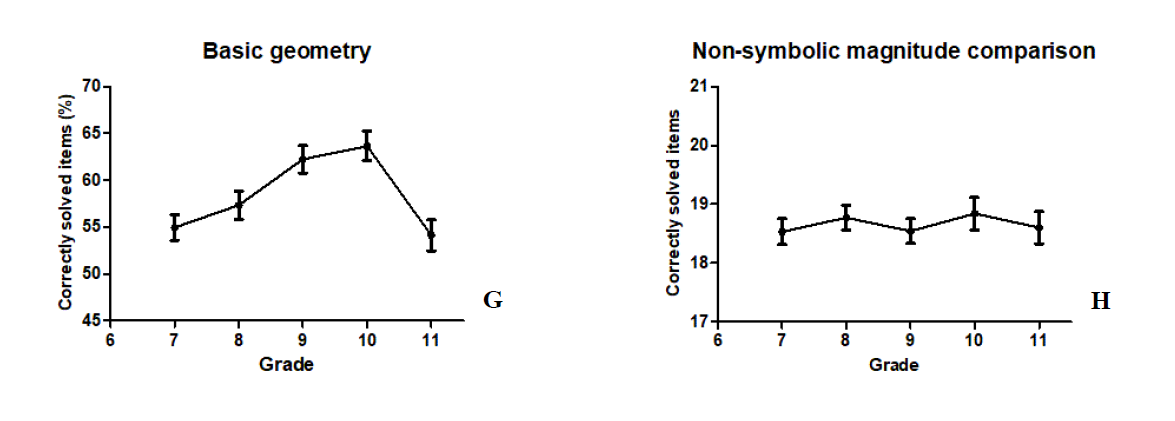


**Figure A4:** Students’ performance for basic geometry **(G)** and non-symbolic magnitude comparison **(H)** scales distinguished by grade levels (7^th^ grade *N* = 200, 8^th^ grade *N* = 215, 9^th^ grade *N* = 210, 10^th^ grade *N* = 136, 11^th^ grade *N* = 178).


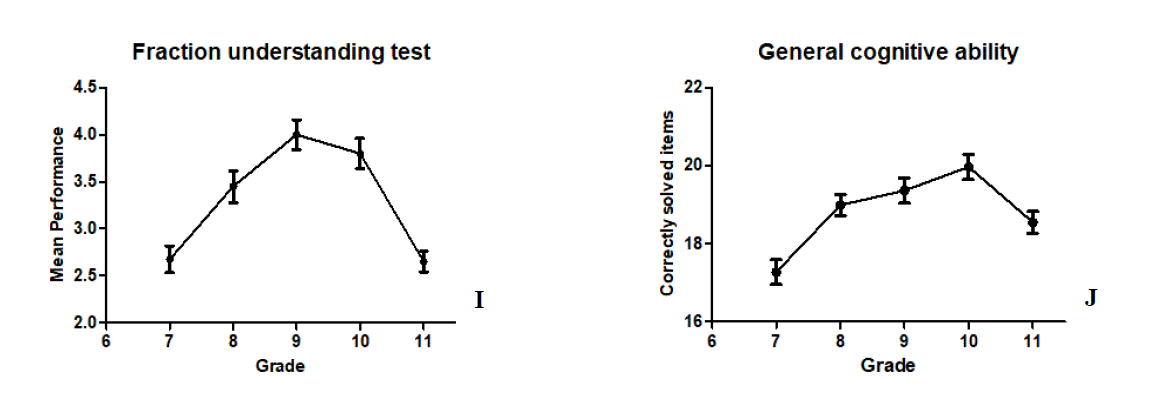


**Figure A5:** Students’ mean performance for fraction processing test **(I)** and performance for general cognitive ability **(J)** scales distinguished by grade levels (7^th^ grade *N* = 200, 8^th^ grade *N* = 215, 9^th^ grade *N* = 210, 10^th^ grade *N* = 136, 11^th^ grade *N* = 178).
